# The role of the amyloid precursor protein (APP) on tau phosphorylation and aggregation in Alzheimer’s Disease

**DOI:** 10.1002/alz.095493

**Published:** 2025-01-09

**Authors:** Annetta Yuwono, Thais Rafael Guimaraes, Amantha Thathiah

**Affiliations:** ^1^ University of Pittsburgh, Pittsburgh, PA USA

## Abstract

**Background:**

Alzheimer’s Disease (AD) is characterized by the accumulation of amyloid‐β (Aβ) plaques and neurofibrillary tangles (NFTs). NFT formation is due to the progressive pathological accumulation and aggregation of the microtubule‐associated protein tau (MAPT) in degenerating neurons. Intriguingly, the amyloid precursor protein (APP), which is cleaved to generate Aβ, is specifically upregulated in NFT‐bearing neurons. Moreover, familial mutations in *APP*, but not *MAPT*, play a causal role in AD development. Although APP pathobiology is undoubtably involved in AD progression, the lack of appropriate models has hindered our understanding of whether APP directly impacts tau aggregation in AD.

**Method:**

We investigated the involvement of APP in tau aggregation and phosphorylation using HEK293 cells that overexpress full‐length APP and a novel optogenetic system to induce tau aggregation, termed optoTAU. We utilized biochemical and immunocytochemical analysis of total tau, phosphorylated tau (pTau), APP, and Aβ species to establish a causal role and investigate the putative mechanism via which APP influences tau pathogenesis.

**Result:**

First, we show that overexpression of APP in optoTAU expressing cells increases tau aggregation, favoring increases in pTau levels. Despite a known relationship between Aβ generation and pTau formation, we show that treatment with α‐, β‐, and γ‐secretase inhibitors does not reverse APP‐mediated tau aggregation. As such, we investigated Aβ‐independent mechanisms through which APP may influence tau aggregation. We show that tau and APP directly interact, and this interaction is enhanced in cells undergoing tau aggregation. Furthermore, APP‐bound tau is not bound to tubulin and aggregated tau displays reduced co‐localization with tubulin, suggesting a putative effect of tau aggregation in disrupting tau physiological interactions. Lastly, we show that APP overexpression enhances cell toxicity in the presence of tau aggregation, which could further potentiate neuronal death in AD brains.

**Conclusion:**

Our results elucidate a direct impact of APP on tau aggregation and confirm the synergistic effect of APP and tau in AD pathogenesis. Future studies aimed at harnessing this relationship in patient‐derived neurons will continue to dissect APP‐dependent mechanisms upstream of tau pathogenesis.

